# Recent applications of pharmacometrics and systems pharmacology approaches to improve and optimize drug therapy for pregnant and lactating women

**DOI:** 10.1002/psp4.13269

**Published:** 2024-11-18

**Authors:** Priya Jayachandran, Jane Knöchel, Brian Cicali, Karen Rowland Yeo

**Affiliations:** ^1^ Regeneron Pharmaceuticals, Inc. Tarrytown New York USA; ^2^ Institute of Mathematics University of Potsdam Potsdam Germany; ^3^ Department of Pharmaceutics, Center for Pharmacometrics and Systems Pharmacology, College of Pharmacy University of Florida Orlando Florida USA; ^4^ Certara UK Limited (CPT Division) Sheffield UK

Drug exposure to a fetus during pregnancy or an infant during breastfeeding remains a key concern for women of reproductive age, and this risk potential has led to the exclusion or under‐representation of pregnant and lactating women in clinical trials. When included, studies have typically been underpowered or key biomarkers have been omitted. Ideally, robust data on drug exposure in mothers, fetuses, and breastfeeding infants are required to perform appropriate safety and efficacy assessments to make informed decisions regarding medication use in pregnant and lactating women. The US Food and Drug Administration (FDA) and the International Council of Harmonization (ICH) have recently released initiatives such as the Diversity Action Plan (DAP) (https://www.fda.gov/media/179593/download) and the *E21 Efficacy Guidelines for Inclusion of Pregnant and Breastfeeding Individuals in Clinical Trials* (https://database.ich.org/sites/default/files/ICH_E21_Final_Concept_Paper_2023_1106_MCApproved.pdf), which are changing the frontiers of inclusion. These regulatory initiatives are providing the impetus for the conduct of more clinical pregnancy and lactation studies by pharmaceutical companies. While the ethical, operational, enrollment, and study design challenges in study conduct are significant, they offer an opportunity for pharmacometrics and systems pharmacology (PSP) to play a key role in making clinical studies more inclusive and supporting clinical data to inform the drug label. This themed issue in *CPT: Pharmacometrics and Systems Pharmacology* on pregnancy and lactation offers perspectives on regulatory drivers for drug research in pregnant and lactating women, improves our understanding of non‐clinical safety data to inform drug exposure in lactation, and spotlights recent quantitative applications in pharmacometrics and physiologically‐based pharmacokinetic (PBPK) modeling to optimize drug therapy for pregnant and lactating women.

## REGULATORY DRIVERS FOR DRUG RESEARCH IN PREGNANT AND LACTATING WOMEN

In 2022, the FDA published the draft *Diversity Plans to Improve Enrollment of Participants from Underrepresented Racial and Ethnic Populations in Clinical Trials Guidance for Industry* (https://www.fda.gov/media/179593/download). While emphasizing race and ethnicity, the FDA encouraged sponsors also to submit plans for other underrepresented populations defined by pregnancy and lactation status. This year, the draft guidance was superseded by the draft *Diversity Action Plans to Improve Enrollment of Participants from Underrepresented Populations in Clinical Studies*, which calls to action improved enrollment of participants from underrepresented populations in clinical studies. Complementary to the FDA DAP, the ICH released the E21 final concept paper (2023) focusing on a global framework and best practices for inclusion of pregnant and lactating women in clinical trials.

The ICH E21 guideline uses the ICH E11 guidance for pediatrics as its foundation. In their perspective, Coppola et al.^1^ offer insights into how the pediatric development extrapolation framework described in the ICH E11 guidance can be adapted to pregnancy. Using a clinical pharmacology extrapolation framework that utilizes quantitative approaches such as PBPK and population PK (Pop‐PK) modeling, a pregnancy drug development strategy may be built that integrates preclinical and clinical PK, safety, efficacy, potential drug–drug interactions (DDI), post‐marketing and Read‐World Data (RWD). The totality of data would be used to inform decisions on the need for studies, study design and labeling updates, and new data generation to fill existing knowledge gaps. Dallman et al.^2^ further describe the “largely untapped potential” of model‐informed drug development (MIDD) tools including quantitative systems pharmacology (QSP), PBPK, RWD, toxicology modeling, and machine learning to enhance inclusivity in clinical trials. They emphasize the importance of early engagement between regulatory agencies and pharmaceutical companies during drug development to include more pregnant individuals. Manolis et al.^3^ in their perspective from the European Medicines Agency (EMA) indicate that MIDD has the potential to complement clinical evidence, potentially accelerating actionable labeling information for medicine use in pregnancy and lactation as well as increasing confidence in enrolling these individuals in clinical trials. Interestingly, while the EMA recognizes the potential of PBPK as the “tool of choice,” they state that there is limited regulatory experience in this area and that “current uncertainties with PBPK in pregnancy and lactation impede their unconditional use and regulatory acceptance.”

## NON‐CLINICAL SAFETY DATA TO INFORM DRUG EXPOSURE IN LACTATION

During drug development, safety data that typically inform the use of medicines during lactation (and pregnancy) fall into three categories: animal experiments, use of predictive tools to estimate fetal exposures and the partitioning of a drug into the breastmilk, and clinical safety data. Several models to predict the milk‐to‐plasma concentration ratio (M/P ratio) of a drug based on the physicochemical characteristics of the drug (e.g., log P, molecular weight, plasma protein binding) are available and undergoing assessment. Although some of these models have evolved to reflect the changing drug space (more metabolically stable drugs susceptible to transporter‐mediated uptake and efflux), they are not yet considered sufficiently robust for prospective prediction of drug concentrations in human milk, especially if transporter‐mediated secretion is involved. These predictive algorithms can be integrated within a PBPK framework to estimate and understand the transfer of drugs into breast milk as well as identify drugs that may require clinical lactation studies.

The systematic review by Gong et al.^4^ is timely as it identifies the key mechanisms involved in the transport of drugs to breastmilk (passive transport, active transport, lipid co‐transport, and transcytosis), as well as 20 transporters that are either up‐ or down‐regulated during lactation. Similarly, the review by Sychterz et al.^5^ importantly examines the role of the breast cancer resistance protein (BCRP) in lactation, providing a comprehensive overview of the current evidence of its role in lactating animals and humans (mothers and infants). The authors highlight a potential noninvasive biomarker (riboflavin) for assessment of BCRP‐mediated activity, and the utility of liquid biopsy to elucidate and enhance the understanding of this transporter as well as to parameterize PBPK models in lactation.

The usefulness of predictive algorithms for M/P ratios within a PBPK framework for lactation is explained in detail in a tutorial by Pansari et al.^6^ Case studies demonstrate applications of the approach to inform and support clinical lactation studies by assessing untested scenarios (impact of colostrum, foremilk versus hindmilk, potential pH changes on the M/P ratio). Cole et al.^7^ used a similar approach to assess if this method is suitable to determine the exposure of the low solubility/low bioavailability drug, albendazole, and its metabolite, in breast milk. The M/P ratio was well predicted for the metabolite but not the parent drug itself, which is highly lipophilic, a finding, which has significance for other drugs with similar properties. Humerickhouse et al.^8^ also use a lactation modeling workflow for pregabalin, which is mainly renally excreted. The authors emphasize the need to obtain both pediatric and lactation data and integrate them into a PBPK modeling platform to further enhance our understanding of infant drug exposure through breastfeeding.

## QUANTITATIVE APPROACHES TO PREDICT DRUG EXPOSURE: POPULATION PK, PBPK, AND MODEL‐BASED META ANALYSIS

Other articles in this themed issue can be broadly classified into two categories: (1) application of core/standard quantitative clinical pharmacology applications including PBPK and Pop‐PK modeling and (2) newer quantitative approaches such as model‐based meta analyses (MBMA) to predict drug exposures in pregnant and lactating women and potentially inform dosing.

Ning et al.^9^ present a PBPK model that captures observed exposures of dolutegravir (UGT1A1 substrate) in non‐pregnant and pregnant healthy volunteers, the umbilical cord, lactating mothers, and breastfed neonates. Interestingly, data from an ex vivo placental perfusion experiment were integrated within the feto–maternal PBPK model to parameterize the dolutegravir transplacental passage. Based on simulations, the authors provide recommendations that support the safe and effective use of dolutegravir in mothers living with HIV. Similarly, fostemsavir is a prodrug of temsavir and is approved in combination with other antiretrovirals to treat HIV infection. Without adequate treatment, HIV transmission to the fetus can occur during pregnancy, labor and birth, or through breastfeeding. Salem et al.^10^ present a PBPK model that demonstrates no dose adjustment is needed for fostemsavir during pregnancy. In both cases, drug exposure in the fetus, breastfed neonates, and infants in the context of prophylactic coverage or the potential to select for viral resistance was considered. Co‐infection of HIV and tuberculosis is associated with poor health outcomes for mothers and infants and necessitates treatment during pregnancy. Atoyebi et al.^11^ leveraged a previously published PBPK model developed to elucidate the DDI between atazanavir boosted with ritonavir (ATV/r) and rifampicin, to investigate dosing strategies that can overcome the DDI effect during pregnancy considering pregnancy‐induced biological changes.

Pregnant women with opioid use disorder face a significant health risk. While the benefits of naltrexone as an effective treatment option have been recognized, there are a number of issues associated with the oral formulation, including extensive first‐pass metabolism. Shenkoya et al.^12^ use clinical data from a pregnancy study involving oral naltrexone and a PBPK model for naltrexone to bridge across different routes of administration and provide dose recommendations for a newly approved extended‐release injectable naltrexone to effectively manage opioid use disorder during pregnancy.

Although PBPK modeling has been typically used for simulations in pregnant and lactating women, an increasing number of publications are based on pharmacometric approaches. Menshykau et al.^13^ use a frequentist prior approach, leveraging an existing Pop‐PK model for certolizumab pegol in non‐pregnant adult patients, to model the PK in pregnant women with chronic inflammatory diseases. Their analysis compared exposures between pregnant and non‐pregnant women to determine whether a dose adjustment was warranted for women during pregnancy. Willeford et al.^14^ present a target‐mediated drug disposition (TMDD) model that characterizes subcutaneously administered monoclonal antibody PK in pregnancy using time‐dependent changes in body weight and central volume and drug‐specific target engagement information. The authors used the model to recommend an optimal dosing regimen that maintains drug exposure above a target level for a phase II study in pregnant women.

Chen et al.^15^ provide a novel use of MBMA to establish the dose–response relationship for combined oral contraceptives (progestins with ethinyl estradiol) with breakthrough bleeding, a pharmacodynamic end point known to contribute to non‐adherence and discontinuation of combined oral contraceptives resulting in unintended pregnancies. The resulting model can be used to support optimal dosing regimens and evaluate clinically relevant factors on breakthrough bleeding.

## GLOBAL EXCHANGE OF CLINICAL DEVELOPMENT STRATEGIES FOR PREGNANT AND LACTATING WOMEN AND THEIR INFANTS

Classical examples of clinical trial designs, particularly PK studies, in pregnant and lactating women can be found where prevention of disease transmission from mother‐to‐child (perinatal) is a high priority. Infectious diseases, such as HIV, malaria, and tuberculosis are a leading source of perinatal disease, with high maternal infection and mortality rates, especially in low‐ and middle‐income countries (LMICs). Examples of progress to improve clinical development strategies and practices for pregnant and lactating women in LMICs using PSP can be applied globally, irrespective of population or trial site location. This themed issue specifically sought to spotlight these examples from authors located in LMIC to demonstrate knowledge exchange and scientific perspectives on quantitative clinical pharmacology across the global community.

Using an initial study design informed by stochastic simulation and estimation and with limited prior information about drug exposure in breast milk for the anti‐mycobacterial drug rifampicin, Kawuma et al.^16^ provide a successful example of how interim analyses can be incorporated into a protocol for an observational PK study with lactating mother–infant pairs; the analysis was used to define the transfer of rifampicin to breastfed infants and quantify drug exposure in maternal plasma, breast milk, and infant plasma. Paired plasma‐breast milk PK data obtained from an observational PK study were used by Ojara et al.^17^ to characterize drug transfer from maternal plasma to breast milk for lamivudine, an antiretroviral used to treat perinatal HIV. An infant's daily dose of lamivudine was calculated using estimated breast milk concentrations and the breast M/P ratio. The modeling framework for characterizing lactation PK can be readily extended to other drugs. Ding et al.^18^ characterized the PK of amodiaquine and piperaquine, artemisinin‐based combination therapy used first‐line for the treatment of malaria, in pregnant women across their second and third trimesters of pregnancy. Their analysis compared exposures between pregnant and non‐pregnant women to determine whether a dose adjustment was warranted for women in their second and third trimesters of pregnancy.

In conclusion, this themed issue on pregnancy and lactation presents recent quantitative applications of PBPK modeling and pharmacometrics in drug development, clinical, and global health settings. Interestingly, PBPK modeling, typically used as the quantitative method of choice for drug research in pregnancy, appears to have shifted application from pregnancy to lactation. An increasing number of Pop‐PK modeling applications that seek to improve clinical development strategies is also encouraging. Eke et al.^19^ advocate for a hybrid modeling approach that combines the feto–maternal biological system parameters integrated with PBPK models with the large‐scale variability captured in Pop‐PK models; bridging maternal and fetal pharmacology learnings may generate more accurate predictions of drug exposure during pregnancy. Applications of more mechanistic models (e.g., QSP) and novel quantitative methods (e.g., RWD, artificial intelligence, and machine learning), all of which are essential MIDD tools, remain relatively underused presently, offering an area for advancement. Furthermore, while the primary focus of articles in this issue was small molecules, the increasing emergence of novel therapeutic modalities in drug development may warrant extensions or convergences of existing models and methods. Increasing regulation to make clinical studies more inclusive, especially through early engagement between regulators and sponsors, is likely to accelerate the application of MIDD across drug development to support and improve clinical trial design, inform dose selection, and optimize drug therapy for pregnant and lactating women. Successful engagement will facilitate equitable healthcare in maternal‐fetal medicine in the years ahead; it remains the responsibility of all disciplines that participate in drug development, from clinicians to regulators to statisticians to clinical and quantitative pharmacologists.

## FUNDING INFORMATION

No funding was received for this work.

## CONFLICT OF INTEREST STATEMENT

The authors declared no competing interests for this work.
